# The Synthesis of carbon dots//zincoxide (CDs/ZnO-H400) by using hydrothermal methods for degradation of ofloxacin antibiotics and reactive red azo dye (RR141)

**DOI:** 10.1038/s41598-024-53083-3

**Published:** 2024-01-30

**Authors:** David Nugroho, Khemika Wannakan, Suwat Nanan, Rachadaporn Benchawattananon

**Affiliations:** 1 Department of Integrated Science, Faculty of Science, Khon Kaen University, Khon Kaen, 40002 Thailand; 2 Materials Chemistry Research Center, Department of Chemistry and Center of Excellence for Innovation in Chemistry (PERCH-CIC), Faculty of Science, Khon Kaen University, Khon Kaen, 40002 Thailand

**Keywords:** Photocatalysis, Photocatalysis

## Abstract

The development of photocatalytic powders to remove contaminants from air solutions is an important field of research in the field of environmental conservation. CD/ZnO-H400, a heterogeneous photocatalytic production, is utilized to degrade the reactive red dye and the antibiotic ofloxacin found in wastewater. This study explains the synthesis of carbon dots (CDs) derived from coconut air and zinc oxide (ZnO) using a hydrothermal method at a temperature of 180 °C with a duration of 4 h and subsequently calcinated at a 400 °C temperature for 4 h. This shows a significant improvement in photocatalytic performance due to improved delivery efficiency at the interface. The cost-efficient use of solar energy allows the comprehensive elimination of harmful pollutants through detoxification. The removal of the contaminant takes place through the first-order reaction, with RR141 showing the highest constant rate at 0.03 min^−1^, while ofloxacin has a constant speed at 0.01 min^−1^. The photocatalytic stability is measured after five cycles. The study also tested the impact of sunlight on degradation, showing a degrading rate of 98% for RR141 and 96% for ofloxacin. This study displays a new catalyst powder synthesized from carbon dots derived from the air, coconut and ZnO, showing remarkable photoactivity to completely remove harmful dyes and antibiotics from the surrounding environment.

## Introduction

According to recent research, industrial wastewater is found to contain a high concentration of azo dyes (RR141) and the chemical structure of the dye is typically resistant to changes^1–7^. The presence of several hazardous chemicals that have been attributed to the degradation of the dye is not fully complete, as reported in previous studies^5,8^. Hence, it is crucial to promptly eliminate harmful colors from freshwater sources. Fluoroquinolone-based antibiotics have been predominantly employed in the control of bacterial infections for an extended period of time. Following their usage, these antibiotics were released into the aquatic environment^6,9^. It has been discovered that living organisms do not fully metabolize antibiotics. Furthermore, it should be noted that the drugs in question lack biodegradability, leading to the accumulation of antibiotic residues in water. As a result, it is imperative to ensure the complete degradation of the pollutant^10–13^.

Semiconductor photocatalysis has garnered significant research attention as a means of purifying water and air, in contrast to other conventional pollutant treatment methods. The utilization of clean technology in the preparation of photocatalyst nanostructures has led to the observation of sample agglomeration in cases where crystal growth cannot be effectively controlled. In order to address the matter at hand, several capping agents were implemented in the preparatory phase to regulate both the morphology and photocatalytic characteristics of the catalysts^14–17^. The ZnO photocatalyst has gained significant attention owing to its exceptional transport characteristics, economical production cost, and adaptable morphology^18,19^. The practical application of ZnO is limited by the primary factors of poor sunlight performance and the occurrence of photocorrosion. Various methods have been employed for the synthesis of ZnO, as presented in Table 1 based on the existing literature^18–22^. Semiconducting nanomaterials were synthesized via a chemical precipitation technique, which offers the benefits of cost-effectiveness, simplicity, and controllability. Theoretically speaking, the photocatalytic efficiency of ZnO, which is UV-active, is hindered by inadequate separation of electron–hole pairs. The enhancement of ZnO's inherent properties has been achieved through two methods, namely doping or decorating the material and constructing a heterostructure^11,23–28^. It is anticipated that the dispersion of noble metals selected onto ZnO will result in increased absorption of visible light. The outcome is attributed to the surface plasmon resonance effect of the noble metal^29–31^. Furthermore, the duration of charge generated by the photo is prolonged, leading to improved photocatalytic efficacy in ZnO decorated with metal in comparison to unaltered ZnO. The current investigation involved the synthesis of the ZnO photocatalyst in combination with diverse composites^32–38^. The production of ZnO was initially carried out via a chemical precipitation process of a straightforward nature. Subsequently, ZnO is a very attractive candidate in the field of photocatalysis because of its advantageous characteristics, including its non-toxicity, low cost, easy accessibility, and wide range of band-gap, especially in the near-UV–Vis region. ZnO is well-suited for solar-driven photocatalytic applications since it absorbs around 90 to 93% of the total solar energy in this area^39–52^.

**Table 1:** Comparison of RR141 azo dye Ofloxacin antibiotic degradation using various photocatalysts.

Catalyst	Concentration	Catalyst amount	Light	Lamp	Time (min)	Photodegradation (%)	Ref.
Photodegradation RR141							
SDS-capped ZnO	10 mg	50 mg	Uv	125 W	240	95	^4^
SDS-capped ZnO	10 mg	50 mg	Visible	15 W	240	60	^4^
Bi_4_MoO_9_	10 mg	50 mg	Uv	125 W	240	68	^28^
CDs/ZnO-400	10 mg	50 mg	Uv	125 W	240	98	This work
CDs/ZnO-400	10 mg	50 mg	Solar light	–	240	98	This work
Photodegradation ofloxacin							
BiVO_4_	10 mg	50 mg	Visible	15 W	240	55.5	^33^
BiVO_4_	10 mg	50 mg	Visible	15 W	240	62	^52^
Co_3_O_4_/TiO_2_/GO	10 mg	25 mg	Uv	300 W	90	91	^53^
CDs/ZnO-400	10 mg	50 mg	Uv	125 W	240	99	This work
CDs/ZnO-400	10 mg	50 mg	Solar light	–	240	96	This work

Carbon dots (CDs) are a novel category of carbon-based nanomaterials that possess a three-dimensional nanostructure and dimensions that are smaller than 10 nm^53–56^. CDs possess an sp^3^/sp^2^ hybridised structure, rendering them suitable for utilization in diverse domains such as bioimaging, biosensors, pH sensing, and biomolecule or drug delivery, owing to their fluorescence characteristics^57,58^. CDs have been identified as a viable alternative to nanoclusters and conventional dye molecules in the field of optoelectronics, owing to their adaptability in terms of photoluminescence (PL) emission wavelengths^59,60^. The unique features and favorable attributes of carbon dots have generated significant attention in their possible application in photocatalytic processes. The incorporation of carbon dots into photocatalysis is justified by their remarkable skills in light absorption and charge separation. The combination of carbon dots with conventional photocatalytic materials is advantageous due to their variable photoluminescence and exceptional electron transport capabilities, rendering them very effective as co-catalysts or sensitizers^61,62^. The collaboration between these factors boosts the absorption of light across a wider range of wavelengths and facilitates effective separation of charges, thereby increasing the overall efficiency of photocatalytic activity. The surface engineering and functionalization capabilities of carbon dots offer a versatile framework for customising photocatalytic processes to suit individual applications^63,64^. Carbon dots naturally have the ability to be easily functionalized with a wide range of moieties, such as metals and functional groups. The ecologically friendly characteristics of carbon dots, together with their easily achievable synthesis techniques, enhance their attractiveness in the field of sustainable photocatalysis, Carbon dots also possesses a moderate bandgap energy and exhibits excellent performance under visible light radiation, making it highly significant in terms of its key properties. Consequently, this substance has been previously utilized in catalytic procedures such as photocatalysis, H_2_O separation, CO_2_ reduction, including harmful and dangerous materials^65–67^.

We synthesized ZnO combined with CD from coconut water using hydrothermal methods in this study. This resulted in a significant improvement in the efficiency of photodegradation, leading to the removal of the antibiotics ofloxacin and RR141 by up to 99% and 98% respectively. This study presents a technique for creating very efficient photocatalysts for the purpose of environmental cleanup by combining CD and ZnO.

## Material and methods

### Material

The coconut water used comes from the species *Cocos nucifera *L. which were collected at the traditional market near Khon kaen university student complex food center in March 2023, ethyl alcohol and zinc oxide and all chemical reagents in this research are analytical grade.

### Methods

#### Synthesis the carbon dots (CDs)

By following Nugroho et al.^14^ in the previous literature, carbon dots were synthesized by using 20 mL of coconut water combined with 20 mL of ethyl alcohol, and the hydrothermal synthesis process was carried out in an oven at 180 °C for 4 h. After synthesis, the product was centrifuged at 10,000×*g* rpm for 10 min. After the process, it was filtered through a 0.22 µm nylon filter membrane and dried in an oven for 24 h.

#### Synthesis CDs/ZnO by hydrothermal methods (CDs/ZnO-H)

10 mL of CDs and mixed with 0.1-g ZnO. The synthesis process was performed by a hydrothermal method, by heating in an oven with temperatures 180 °C and times 4 h After synthesis, dry in the oven for 24 h to become powder.

#### Synthesis CDs/ZnO by precipitation methods (CDs/ZnO-P)

10 mL of CDs and mixed with 0.1-g ZnO. The synthesis process was performed by stirrer methods in the Room temperature for 24 h, after mixing the CDs/ZnO dry in the oven for 24 h to become powder.

#### Synthesis CDs/ZnO-H400 and CDs/ZnO-P400

After synthesized the CDs/ZnO-P and CDs/ZnO-H, 1 g of each powder has been prepared and heated in the 400 °C for 4 h under calcination methods by using close system.

#### Photodegradation of pollutants RR141 azo dye and ofloxacin antibiotics

The photocatalytic activity under UV light (a mercury lamp, 125W), The catalyst's photoactivity was assessed through the degradation of the RR141 azo dye and Ofloxacin antibiotic (OFL Antibiotics). 50 mg of the photocatalyst that had been produced was combined with a pollutant solution that had been prepared with a final volume (200 mL and a concentration of 10 mg L^−1^). The suspension was agitated in a dark room environment for a duration of 60 min to establish an equilibrium state of adsorption–desorption for the photocatalyst. Following a 60 min period under dark conditions for the photo irradiation process, a 5 mL sample was collected and put through centrifugation at 5000 RPM for 10 min. This centrifugation step was performed to obtain the supernatant, which was subsequently analyzed for concentration using the UV–vis spectrophotometric method. The concentration was determined by measuring the absorbance at wavelengths of 544 nm (RR141) and 293 nm (OFL Antibiotics)^1,26,27^, respectively. The photodegradation efficiency was assessed using the equation for photodegradation efficiency, photoactivity (%) = ((1 − C/C_0_) × 100%), which is calculated as the percentage of degradation based on the difference between the initial concentration (C_0_) and the concentration of the pollutant aqueous solution (C) after a given period of irradiation. The degrading response of the pollutant through photocatalysis follows a kinetic model that may be described by the first-order reaction rate equation, ln (C_0_/C) = k1t, where k1 represents the rate constant associated with the first-order reaction. The investigation focused on the impact of several experimental parameters, such as the initial solution pH, photocatalyst loading, and initial pollutant content, on the photoactivity during the examination of dye and antibiotic degradation^1,5,6,9^. In order to investigate the key species responsible for the elimination of the pollutant, individual scavengers (at a concentration of 5 mM) including isopropyl alcohol (IPA), EDTA-2Na, and K_2_Cr_2_O_7_ were introduced in this study. These scavengers were employed to specifically quench hydroxyl radicals, superoxide anion radicals, holes, and electrons, respectively. Furthermore, KI was also employed as a scavenger of surface hydroxyl radicals and holes. The individual scavengers were integrated individually in the presence of the prepared photocatalyst^1,9,68^.

#### Photodegradation of RR141 and ofloxacin antibiotic under sunlight

**Figure 1:**
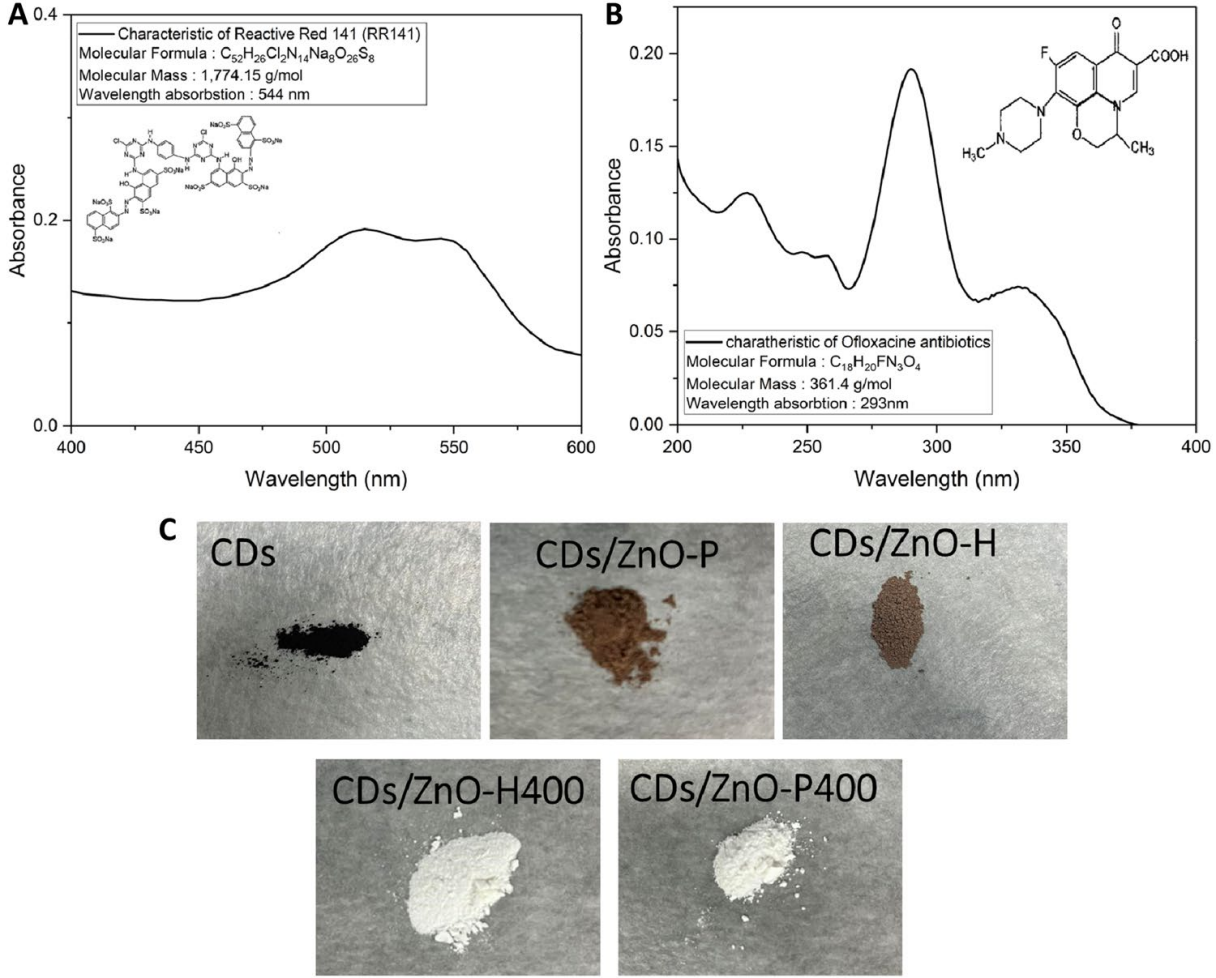
Characteristics of pollutant (**A**) RR141 (**B**) ofloxacin, catalyst powder after synthesized of (**C**).

This study aimed to analyze the photodegradation of RR141 and OFL antibiotics in sunlight. In March 2023, the RR141 (22 March, 11.00 a.m.) and OFL Antibiotics (26 March, 11.00 a.m.) solution conducted sunlight exposure on the rooftop of Science Building Number 04, located inside the Faculty of Science at Khon Kaen University, Thailand. The geographical coordinates of the exposure site were recorded as latitude 16°28′ 33.7″N and longitude 102°49′ 26.2″E (Fig. 1).

## Results and discussions

### Characterization of CDs and various conditions CDs/ZnO

**Figure 2:**
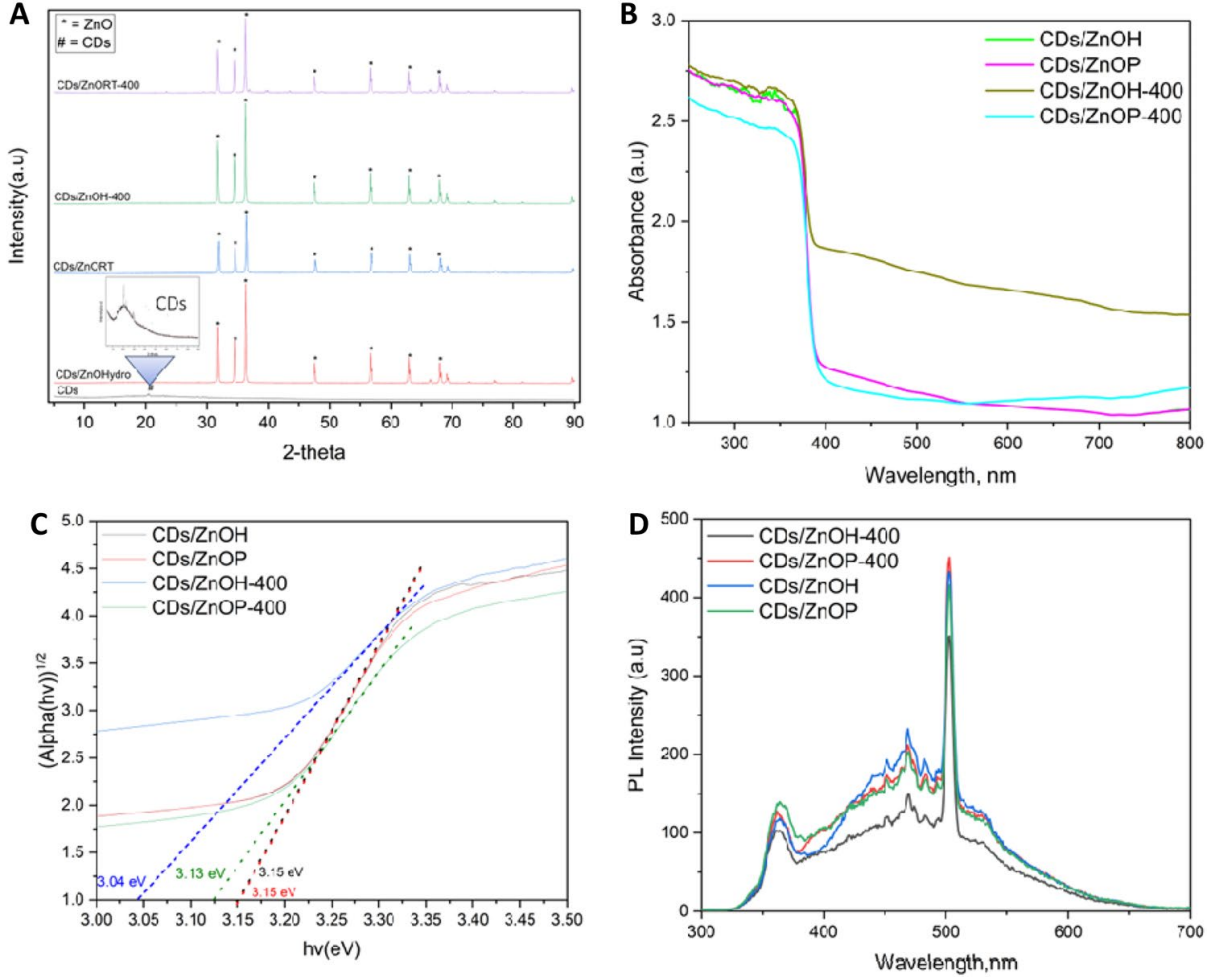
X-Ray diffraction analysis (XRD) of various condition of CDs/ZnO (**A**) diffused reflectance spectra (**B**) Tauc plot of band gap energy (**C**) Fluorescence spectroscopy spectra (**D**).

The XRD Data of CDs/ZnO in the various conditions of synthesis is shows that CDs/ZnO-H, CDs/ZnO-P, CDs/ZnO-H400, and CDs/ZnO-P400 (Fig. 2A) show have a strong crystal peak and shows diffraction peaks of the ZnO at 2θ = 31.7°, 34.6°, 36.2°, 47.7°, 56.6°, 62.7°, and 67.8°, due to (100), (002), (101), (102), (110), (103), and (112), reflection planes, peaks of CDs = 20.8 (002) the crystallites (grains) size from XRD data has been analyst by using formula D = (kλ/β cos θ) and the average of crystallites of CDs, CDs/ZnO-H, CDs/ZnO-P, CDs/ZnO-H400, and CDs/ZnO-P400 is 0.2 nm, 42.3 nm, 37.1 nm, 41.69 nm and 38.9 nm (Table 2). The UV–vis diffuse reflectance spectra of the samples are displayed in Fig. 2B. The Kubelka–Munk formula was applied to figure out the band gap energy of each sample^5^. Figure 2C illustrates this process. Consequently, the absorption edge values of 387 nm were observed in the case of ZnO. The band gap values of bare CDs/ZnO-H, CDs/ZnO-P, CDs/ZnO-H400, and CDs/ZnO-P400 were determined to be 3.16, 3.17, 3.15, and 3.07 eV, respectively. The data indicates that CDs/ZnO synthesized using various methods exhibit a heterojunction structure, as evidenced by the presence of only one energy bandgap that have been show in the result, Based on the photoluminescence (PL) spectra illustrated in Fig. 2D, it can be observed that the CDs/ZnO-H400 photocatalyst exhibited the least intensity in the PL spectra when compared to the other experimental conditions. This observation shows that CDs/ZnO-H400 photocatalyst had the lowest rate of charge carrier recombination.

**Table 2:** Crystallite size of catalyst powder.

Photocatalyst	Peak type	FWHM	Crystal size
CDs	002	32.23	0.20
CDs/ZnO-H	100	0.16	50.80
	002	0.18	47.50
	101	0.16	51.50
	102	0.21	40.69
	110	0.24	37.18
	103	0.27	34.77
	112	0.28	33.64
	Average		42.30
CDs/ZnO-P	100	0.19	64.78
	002	0.20	39.58
	101	0.19	66.12
	102	0.24	52.02
	110	0.27	51.28
	103	0.29	43.40
	112	0.32	44.40
	Average		51.65
CDs/ZnO-H400	100	0.17	47.86
	002	0.17	48.61
	101	0.17	47.94
	102	0.22	38.73
	110	0.25	36.35
	103	0.26	35.57
	112	0.26	36.74
	Average		41.69
CDs/ZnO-P400	100	0.19	43.31
	002	0.20	41.66
	101	0.18	46.51
	102	0.22	39.01
	110	0.26	34.72
	103	0.25	37.27
	112	0.32	29.97
	Average		38.90

**Figure 3:**
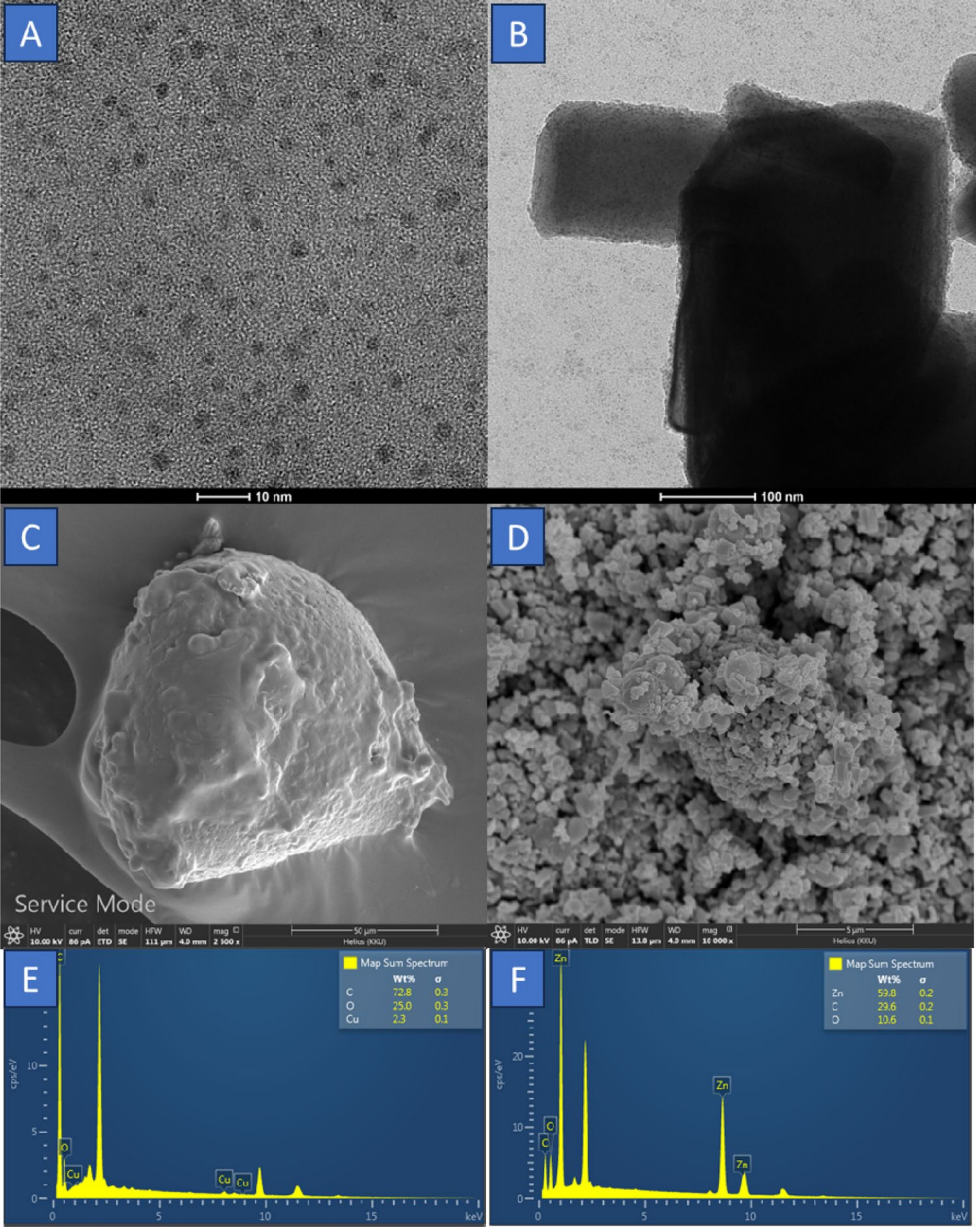
TEM data of (**A**) CDs (**B**) CDs/ZnO-H400, FE-SEM data of (**C**) CDs, (**D**) CDs/ZnO-H400, and EDX of (**E**) CDs (**F**) CDs/ZnO-H400.

**Figure 4:**
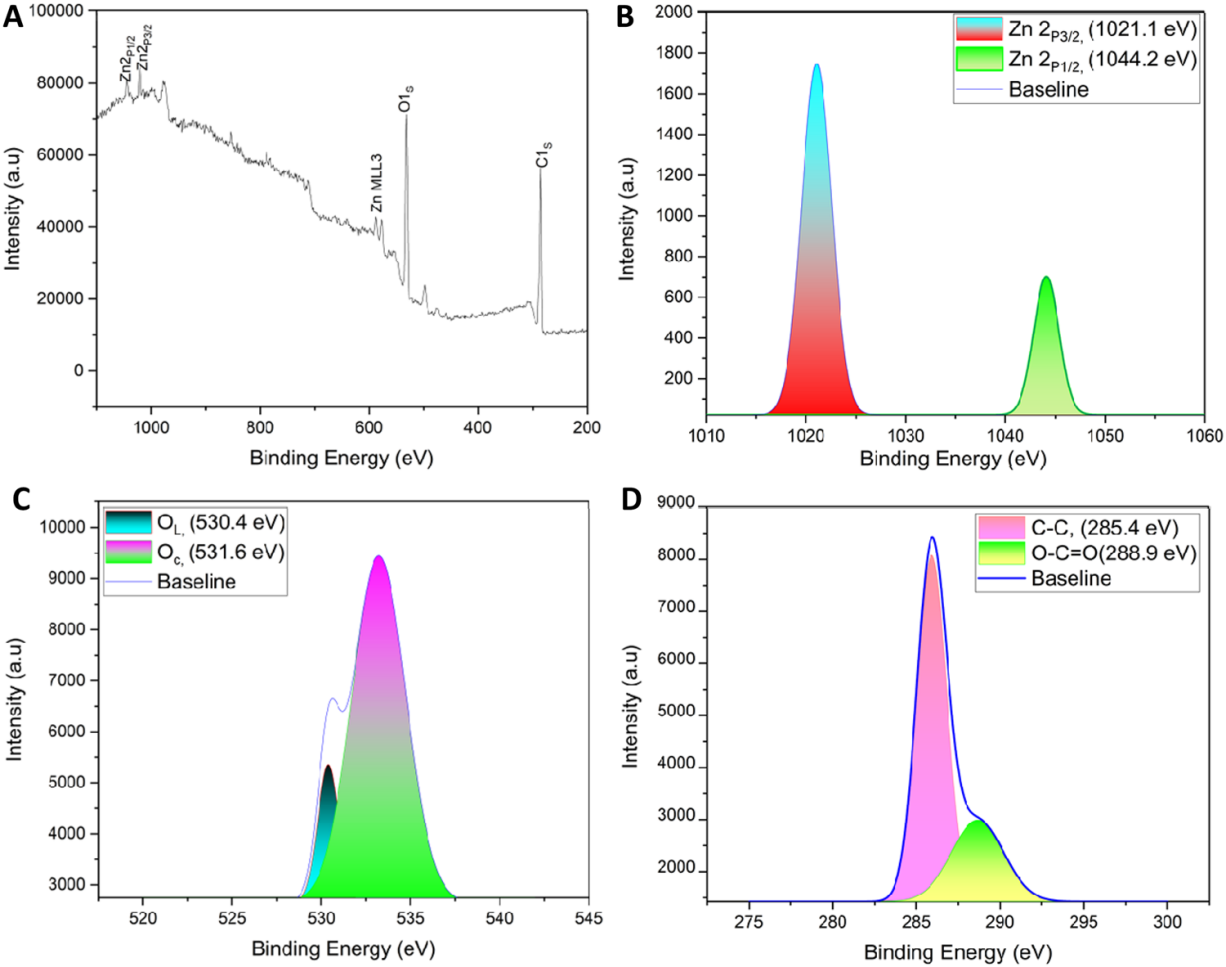
XPS Spectra of data of CDs/ZnO-H400 (**A**) Survey Scan, fine scan of (**B**) Zn 2p (**C**) O 1s, (**D**) C 1s.

The morphological structures of CDs, CDs/ZnO-H400 has been analyst by using transmission electron microscopic (TEM) and field emission scanning electron microscopic (FE-SEM) technique. The FE-SEM of CDs, CDs/ZnO-H400 shown in Fig. 3, CDs from coconut water has the characterization and it’s show that the carbon dots has the characterize is melting in the room temperature (Fig. 3C), but after mixed with the ZnO by using hydrothermal methods, and calcination in the temperature 400 °C and 4 h it’s had unstructured spherical morphology and CDs has been coating onto the ZnO structured that can be seen in Fig. 3D. the particle size of CDs shown in Fig. 3A with the average particles sized of ± 2 nm. Figure 3B illustrates the deposition of ZnO following treatment with CDs, resulting in significantly higher particle size compared to the crystallite size determined through XRD analysis. This increase in size can be attributed to the aggregation of several crystallites. in Fig. 3. In addition, the composition of the CDs/ZnO-H400 and CDs was also analyst for elemental structured by used EDX. The spectrum of the CDs (Fig. 3E) and CDs/ZnO-H400 (Fig. 3F), Fig. 3E indicated the CDs has element existence of Carbon (C), oxygen (O), and copper (Cu) elements with the weight% of C, O and Cu are 72.8%, 25.0% and 2.3%, respectively. The element of CDs/ZnO-H400 shown on Fig. 3F is Zinc (Zn), Carbon (C), and Oxygen (O), with atomic% are 59.8%, 29.6% and 10.6%, respectively. The stoichiometry CDs/ZnO-H400 result of element confirmed that the 2:1 between Zn and C and 5.5:1 atomic ratio of Zn with O. X-ray photoelectron spectroscopy (XPS) was employed to ascertain the elemental composition and chemical oxidation state of CDs/ZnO-H400 at the surface. The spectra that illustrated in Fig. 4. The survey scan spectra depicted in Fig. 4A demonstrate the presence of Zn, O, and C elements inside the CDs/ZnO-H400. The spectra depicted in Fig. 4B performed deconvolution, resulting in the identification of two distinct peaks originating from the pristine ZnO material. These peaks were found to be positioned at 1021.1 eV and 1044.2 eV, corresponding to the Zn 2p3/2 and Zn 2p1/2 states, respectively^65^. From these XPS results we can find out that CDS/ZnO-H400 has a heterostructure elemental composition. The observed difference in binding energies, around 23 eV, suggests the presence of Zn in the ZnO sample in the form of Zn^2+^. The O 1s spectrum displayed in Fig. 4C exhibited a notable level of resolution. Through deconvolution analysis, it was determined that this spectrum consisted of two distinct peaks located at 530.4 eV and 531.6 eV. These peaks were attributed to the presence of adsorbed oxygen originating from the surface hydroxyls^28^. Figure 4D illustrates the resolution of the C 1s spectrum, wherein the data was subjected to deconvolution resulting in the identification of two different peaks at 285.4 eV and 288.9 eV. These peaks can be attributed to the presence of C–C and O–C=O bonds, respectively^51^.

### Photoactivity

The evaluation of the photocatalytic effectiveness of each photocatalyst was performed by observing the photodegradation of the RR141 azo dye and the ofloxacin antibiotic. The concentrations RR141 and OFL antibiotic had measured by utilizing their respective λmax values of 544 nm and 293 nm, respectively^6^. Additionally, an investigation was conducted on the photodegradation process of OFL Antibiotics and RR141 in the presence of sunlight.

#### Photodegradation of RR141 and OFL antibiotics under Uv-light

**Figure 5:**
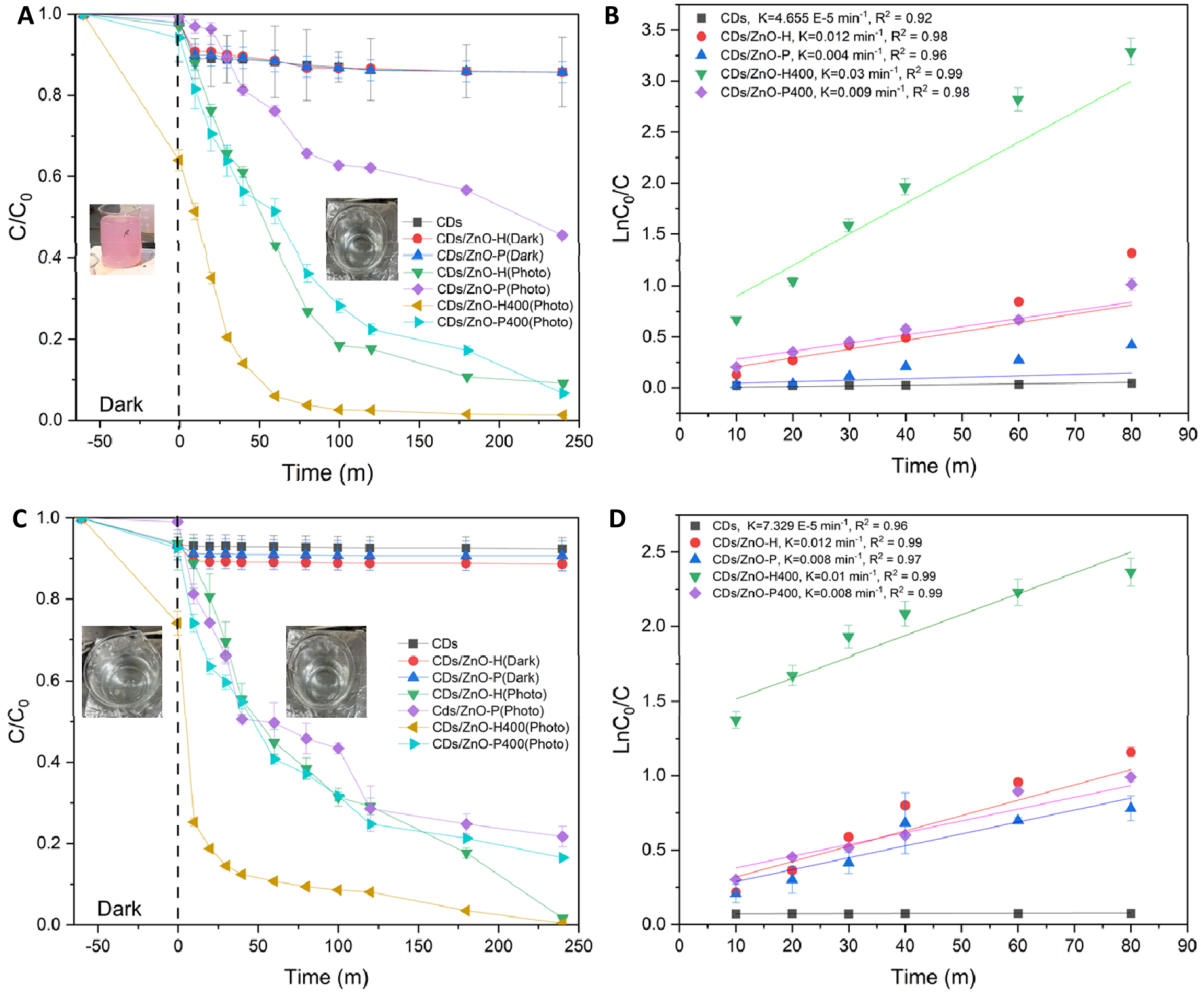
Photodegradation Comparison by used Uv Light in the various condition of CDs/ZnO of RR141 (**A**) C/C_0_ (**B**) LnC_0_/C, Ofloxacin (**C**) C/C_0_ and (**D**) LnC_0_/C (SD, 3 replication).

The analysis includes the examination of the photodegradation of RR141 dye, specifically focusing on the decrease in dye concentration (C/C_0_) over time (t) following exposure to UV light as shown in Fig. 5A, the adsorption technique exhibited a rapid removal rate of the dye RR141. The addition of CDs/ZnO-H, CDs/ZnO-P, CDs/ZnO-H400, and CDs/ZnO-P400 resulted in a decrease in C/C_0_ over time under UV irradiation. Specifically, the application of CDs/ZnO-H400 resulted to a decrease in C/C_0_ from 1 to 0.01 after 240 min, indicating a degradation efficiency of 98% for RR141 when using CDs/ZnO-H400. The nanocomposite exhibited a significant photodegradation efficiency of 98% towards the RR141 dye. The determination of the catalysts' photoactivity was also conducted based on the rate of the photodegradation reaction. The photodegradation rate constant (k) of CDs/ZnO-H, CDs/ZnO-P, CDs/ZnO-H400, and CDs/ZnO-P400 are 0.012, 0.004, 0.03 and 0.009 with the R^2^ is 0.98, 0.96, 0.99, and 0.98. After getting the optimum condition of catalyst powder (CDs/ZnO-H400). The concentration of OFL antibiotics (C/C_0_) after UV light irradiation was examined in order to determine the photodegradation of the antibiotic OFL Antibiotics, the removal of the dye OFL antibiotic via adsorption process has been studied. The integration of CDs/ZnO-H, CDs/ZnO-P, CDs/ZnO-H400, and CDs/ZnO-P400 resulted in a decrease in the C/C_0_ ratio with increasing UV irradiation time (Fig. 5A). Specifically, the application of CDs/ZnO-H400 corresponded to a decrease in the C/C0 ratio from 1 to 0.004 after 240 min. the degradation efficiency of OFL antibiotic by using CDs/ZnO-H400 shows 99%. The ofloxacin was photodegraded 99% in the presence of the nanocomposite. The photodegradation rate constant (k) of CDs/ZnO-H400 shows in the Fig. 5D, photocatalyst provided the UV-driven photodegradation rate constant (k) of 0.01 with R^2^ 0.99.

#### Photodegradation of RR141 and OFL antibiotics under sunlight

**Figure 6:**
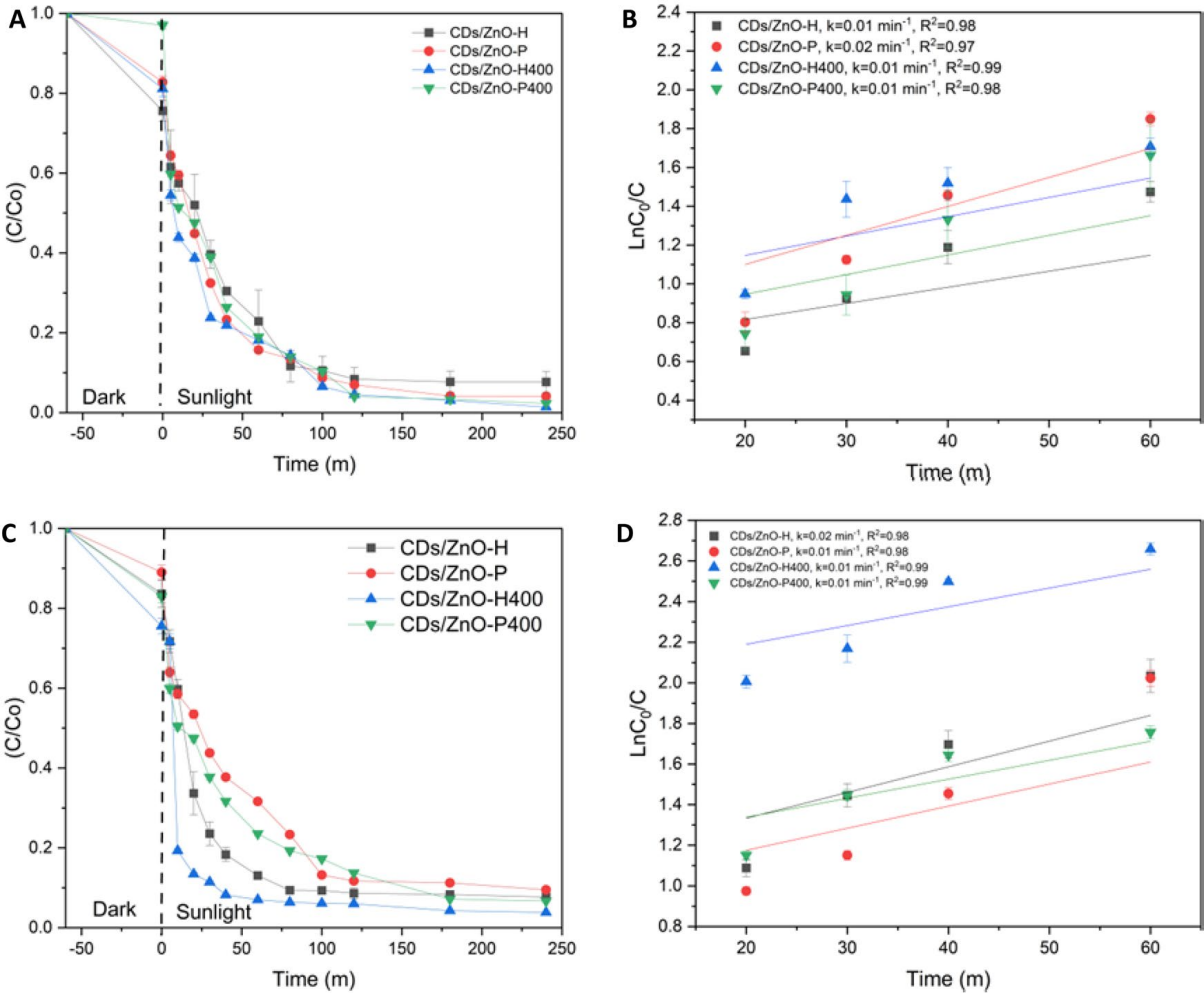
Photodegradation comparison by used sunlight in the various condition of CDs/ZnO of RR141 (**A**) C/C_0_ (**B**) LnC_0_/C, Ofloxacin (**C**) C/C_0_ and (**D**) LnC_0_/C (SD, 3 replication).

The concentration (C/C_0_) of RR141 by uv irradiation under sunlight has been examined in order to determine the photodegradation of the RR141, as shown in Fig. 6, the removal of the dye RR141 is relatively fast. The incorporation of CDs/ZnO-H, CDs/ZnO-P, CDs/ZnO-H400, and CDs/ZnO-P400 resulted in a decrease in the C/C_0_ ratio with increasing UV irradiation time (Fig. 6A), reaching values of 1 and 0.04 after 240 min, respectively. The degradation efficiency of RR141 using CDs/ZnO-H, CDs/ZnO-P, CDs/ZnO-H400, and CDs/ZnO-P400 was found to be 92%, 94%, 98%, and 97%, respectively. The photoactivity of the catalysts was assessed by measuring the rate of the photodegradation reaction. In Fig. 6B, the UV-driven photodegradation rate constants (k) of CDs/ZnO-H, CDs/ZnO-P, CDs/ZnO-H400, and CDs/ZnO-P400 photocatalysts were found to be 0.01, 0.02, 0.01, and 0.01, respectively.

**Figure 7:**
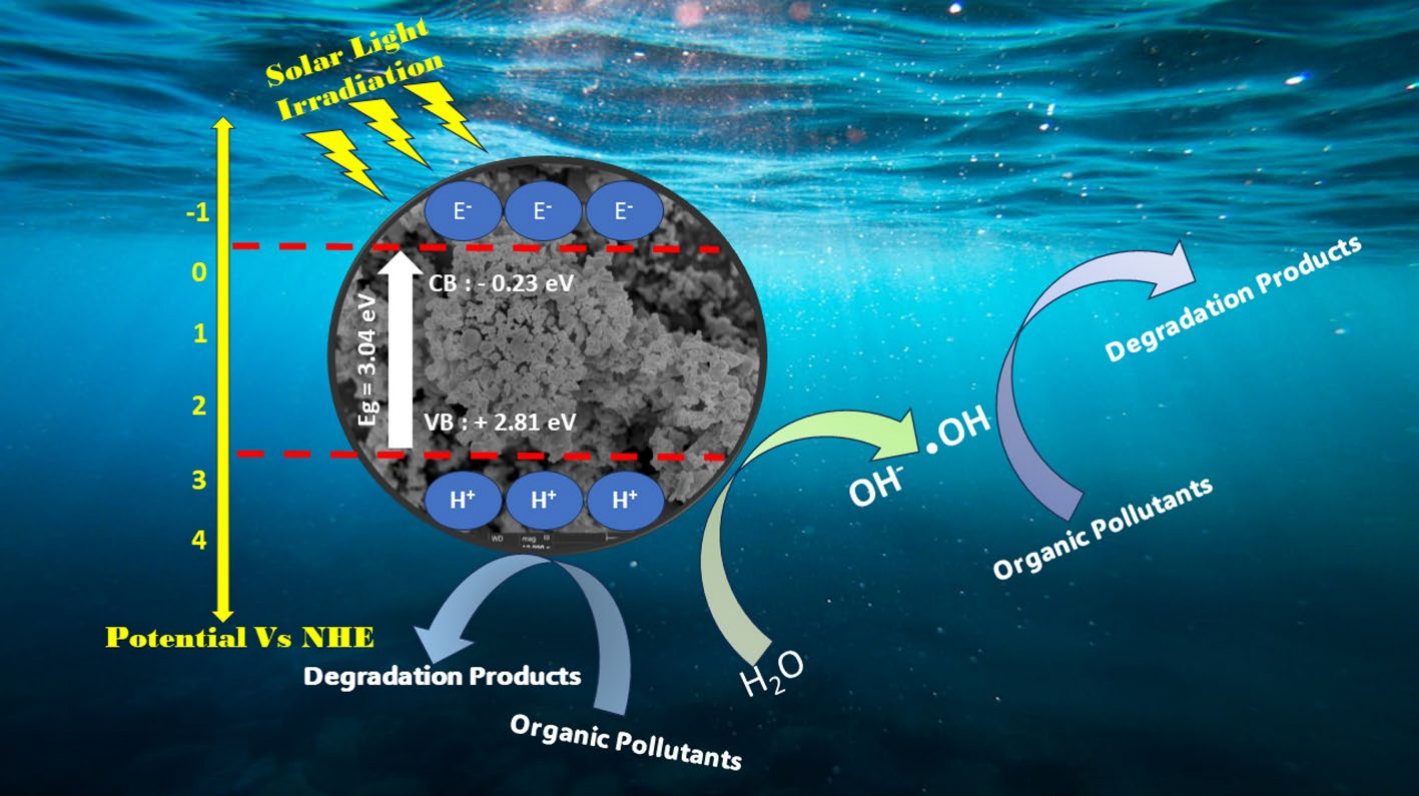
Mechanism scheme photocatalytic degradation of organic pollutants (RR141 and Ofloxacin antibiotics) under solar light irradiation.

The effect of Uv irradiation under sunlight was examined in order to determine the photodegradation of the OFL Antibiotics, as shown in Fig. 6C. In addition, removal of the OFL Antibiotics is moderate, after incorporation of CDs/ZnO-H, CDs/ZnO-P, CDs/ZnO-H400, and CDs/ZnO-P400, lowering of C/C_0_ with UV irradiation time (Fig. 6C) from 1 to 0.07, 0.09, 0.03 and 0.06 respectively, was detected after 240 min. the degradation efficiency of antibiotic OFL Antibiotics by using CDs/ZnO-H, CDs/ZnO-P, CDs/ZnO-H400, and CDs/ZnO-P400 shows 92%, 90%, 96% and 93% it’s mean the optimum condition is by using CDs/ZnO-H400. The determination of the catalysts' photoactivity was also conducted based on the rate of the photodegradation reaction. The photodegradation rate constant (k) of CDs/ZnO-H, CDs/ZnO-P, CDs/ZnO-H400, and CDs/ZnO-P400, in Fig. 5D shown 0.02, 0.01, 0.01 and 0.01 with the R^2^ 0.98,0.98,0.99 and 0.99. The occurrence of electron and hole photogeneration in the conduction band (CB) and valence band (VB), respectively, was observed upon exposure to light irradiation. Subsequently, the generation of reactive species would be anticipated. The determination of the energy levels of the conduction band (CB) and valence band (VB) edges in both Carbon Dots (CDs) and zinc oxide (ZnO) photocatalysts can be achieved by the use of the Mulliken electronegativity theory, (E_VB_ = χ − EC + 0.5_Eg_) and (EC_B_ = E_VB_ − E_g_). E_VB_ represents the valence band potential, EC_B_ denotes the conduction band potential, E_C_ signifies the normal hydrogen electrode potential (about 4.5 eV), and χ represents the absolute value of the electronegativity of the semi-conducting photocatalyst. The electronegativity values of ZnO were determined to be 5.79 electron volts (eV) each. According to the theoretical framework, the valence band (VB) and conduction band (CB) potentials of ZnO were determined to be 2.81 and − 0.23 electron volts (eV), respectively (Fig. 7). In this study, it was observed that the formation of a heterojunction resulted in a measured conduction band potential of ZnO at − 0.22 eV. The band gap energy is measured to be 3.04 electron volts (eV)^1,5,6,28^.

#### The impact of experimental parameters on photoactivity.

**Figure 8:**
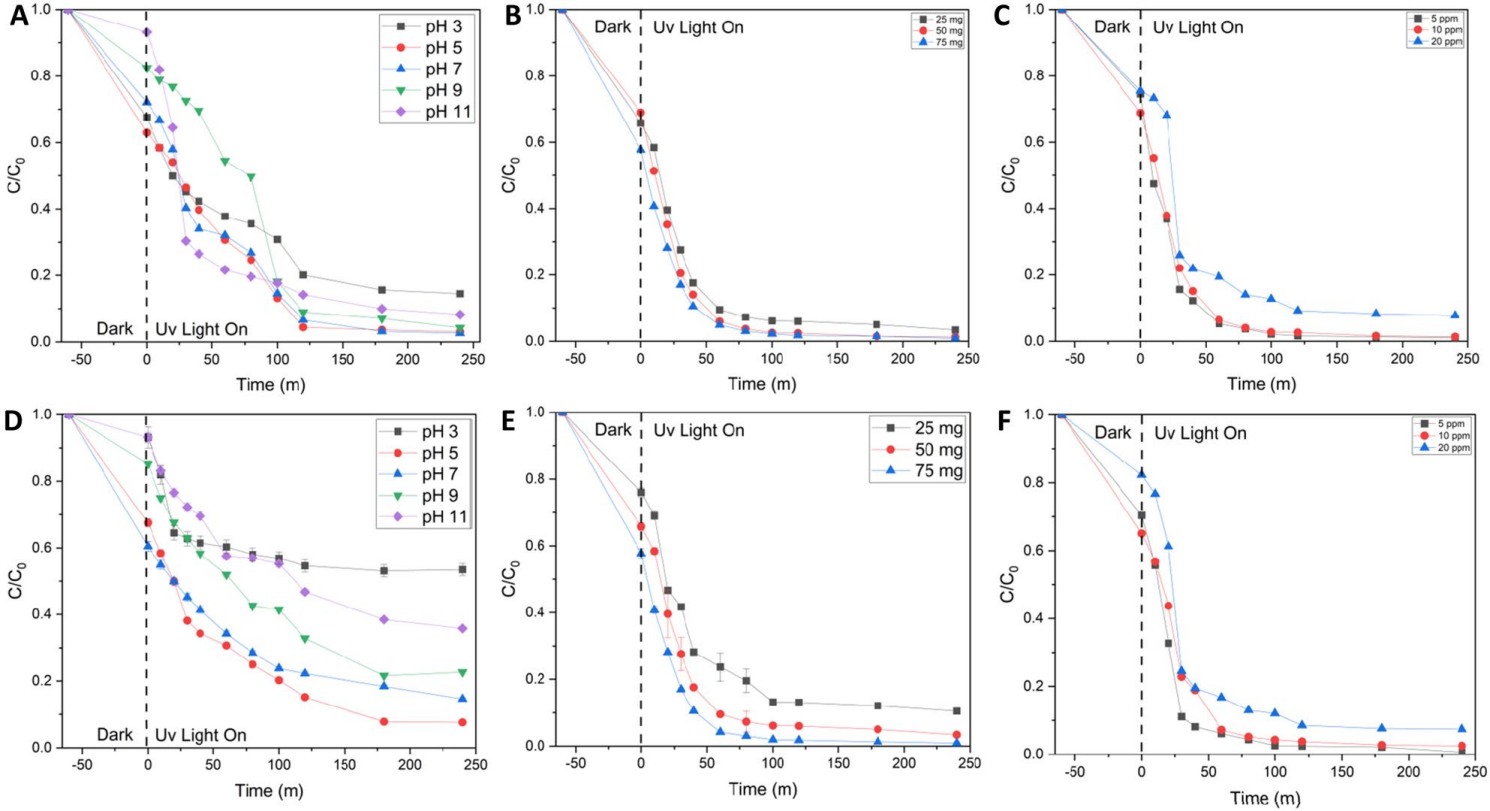
The effect of various conditions of pH (**A**), catalyst concentration (**B**) and (**C**) concentration of RR141, various conditions of pH (**D**), catalyst concentration (**E**) and (**F**) concentration of ofloxacin.

The various conditions to carry out the test at the optimum condition were carried out under various conditions, including the effect of pH, the effect of catalyst concentration, and the effect of concentration of RR141 (Fig. 8). Figure 8A shows that the condition of adding pH7 buffer has optimum conditions compared to the others, with an efficiency of 97.4%. Meanwhile, the optimum condition of various catalyst concentrations is 50 mg (Fig. 8B), and the optimum condition of concentration RR141 is 5 ppm (Fig. 8C). Studying the impact of the experimental factors on OFL antibiotic degradation is shown in Fig. 8D. The pH natural of the antibiotic OFL solution is around 7. Contrarily, in the fundamental pH range between 9 and 11, the anionic OFL antibiotic and the presence of a negative charge on the surface of the photocatalyst cause them to repel one another. a decrease in the extent of adsorption of OFL antibiotics onto the photocatalyst's surface could potentially lead to a decline in the catalytic activity of the photocatalyst. The enhanced adsorption of anionic OFL on the photocatalyst's positively charged surface was ascribed to the heightened photoactivity seen at a pH 5^5,6^. It should be noted that under extremely acidic conditions (pH of roughly 3), the photocatalyst can dissolve. It was also investigated how the photocatalyst content affected the photodegradation of OFL (Fig. 8E). Due to an increase in the number of OFL molecules adsorbed on the photocatalyst's surface and an increase in photocatalyst particle density per unit area of photo irradiation, photoactivity increases as photocatalyst content increases^7^. However, the increase in solution turbidity, which in turn causes a decrease in photo penetration, is what causes the photoactivity to decline at a photocatalyst level of 75 mg. The result will be a decrease in photoactivity. Additionally, it was established how the concentration of OFL affected the photoactivity (Fig. 8F). The performance of the photocatalytic system is decreased by the addition of OFL concentration. The amount of light absorbed by the OFL molecules increases with the concentration of OFL. This could result in a reduction in the amount of light that reaches the surface of the photocatalyst, which would diminish its effectiveness^8^. With 5 ppm of OFL. The produced nanocomposite photocatalyst's photoactivities towards the breakdown of the antibiotic OFL under sunlight were also has been studied^7^.

#### The effect of trapping agent and reusability

**Figure 9:**
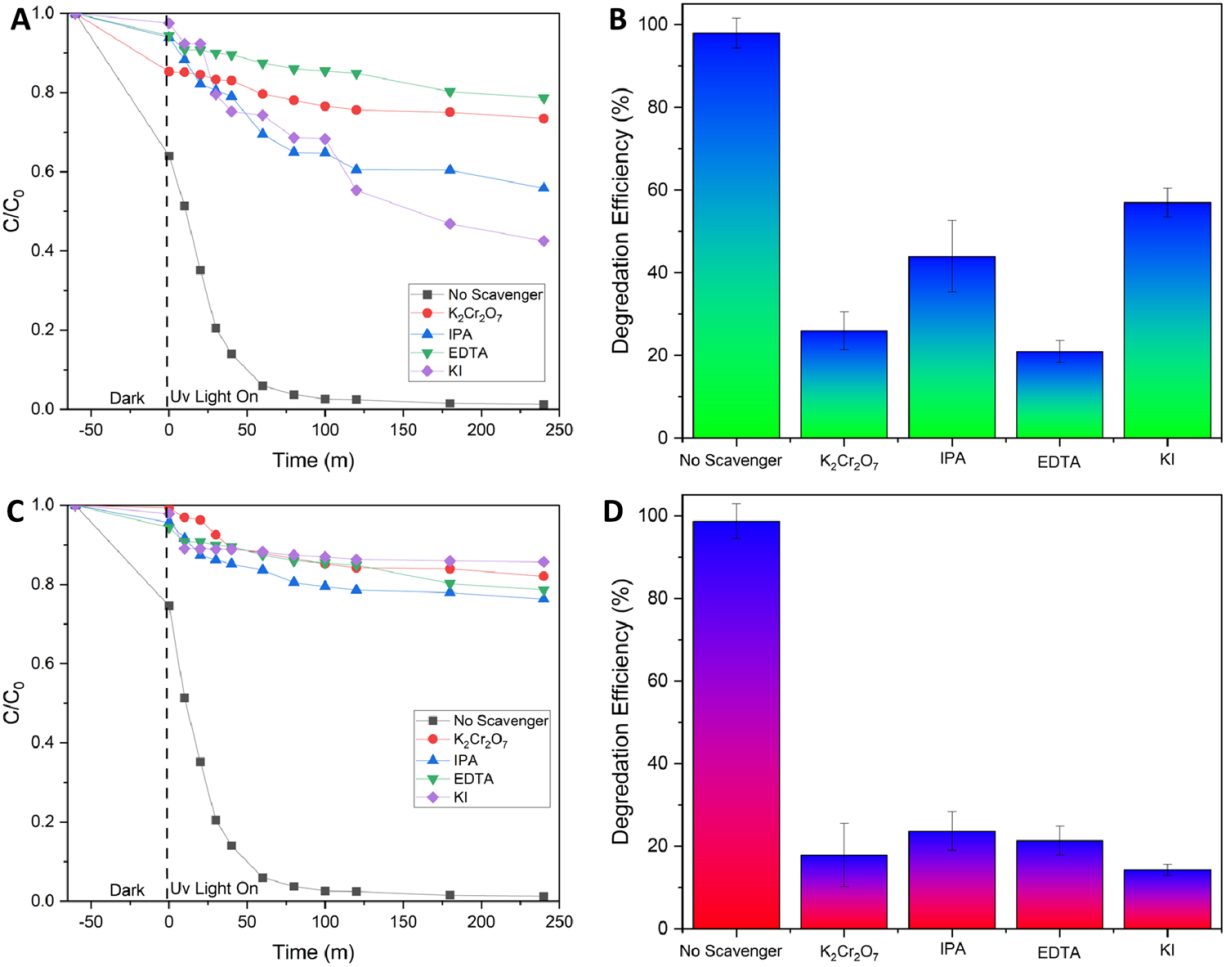
The Concentration (C0/C) by photodegradation on ofloxacin (**A**) the constant rate obtained from degradation of ofloxacin after adding various scavenger (**B**) degradation efficiency on ofloxacin (**C**) The Concentration (C0/C) by photodegradation on RR141 (**D**) the constant rate obtained from degradation of RR141 after adding various scavenger (**E**) degradation efficiency on RR141 (**F**) (SD, 3 replication).

**Figure 10:**
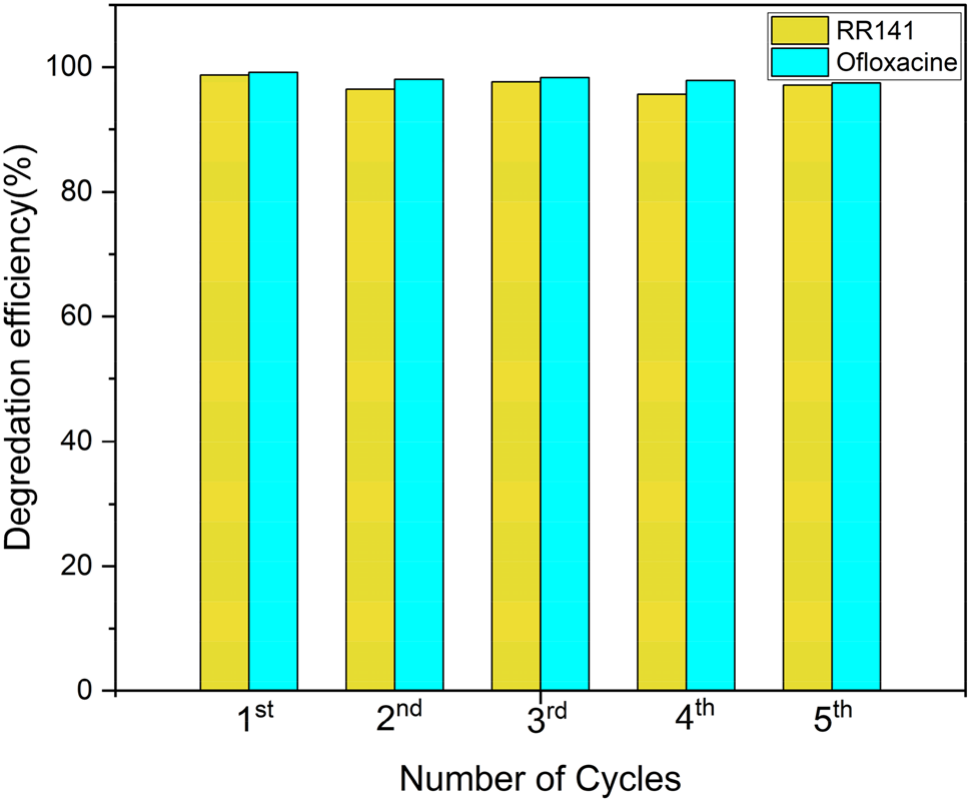
Repetition of photocatalyst toward degradation of RR141 dye and OFL antibiotic by using CDs/ZnO-H400 (a) Uv light (sun light).

**Figure 11:**
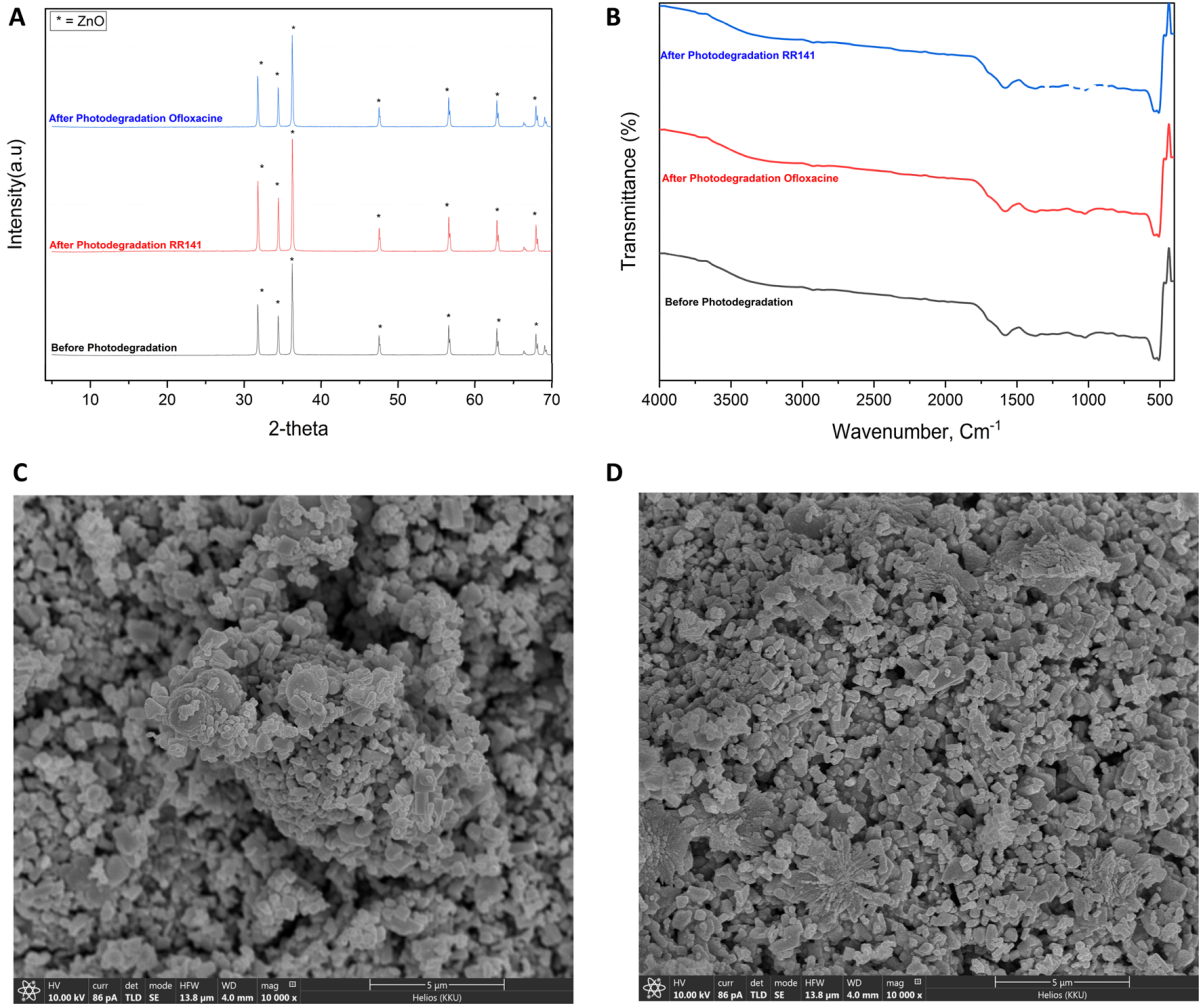
(**A**) XRD of CDs/ZnO-H400 and (**B**) FTIR of CDs/ZnO-H400, FE-SEM of (**C**) CDs/ZnO-H400 after photocat RR141 (**D**) CDs/ZnO-H400 after photocat Oflox.

The trapping experiment by using various trapping agents has been studied for RR141 and OFL Antibiotics. In this study investigated the impact of certain reactive species on the breakdown process of the OFL antibiotic. Specifically, the effectiveness of different scavengers, including EDTA, IPA, K_2_Cr_2_O_7_ and KI were used as a quencher of ⋅OH, h^+^, and e^–^, respectively. Figure 9 illustrates a significant decline in photoactivity subsequent to the photo chat reaction with KI. The efficiency of the reaction was observed to be 14%, accompanied by a notably low rate constant when compared to the absence of a scavenger mechanism. In general, the photogenerated hole is a crucial factor in the process of photodegradation of OFL Antibiotics. Similar to an OFL antibiotic, an OFL Antibiotics antibiotic exhibits comparable characteristics and properties. The efficacy of different scavengers was evaluated in the RR141 experiment. It was observed that the photoactivity significantly decreased following the photo chat interaction with EDTA. The effectiveness of the scavenger process was determined to be 21%, with a notably low corresponding rate constant compared to the absence of any scavenger process.. This investigation also represents a study of the powder's catalytic cycle capabilities. Antibiotic elimination of OFL and RR14 dye was tested in five consecutive trials. After the initial round of photocatalytic investigation, the catalyst underwent the separation process using the whatman 1 paper filter, was cleaned with DI water, and dried in a 60 °C oven for 12 h before being used in the next round until the fifth round. The fifth run of the photocatalyst CDs/ZnO-H400, as illustrated in Fig. 10, demonstrates its remarkable cycling ability. The degradation performance of RR141 by the photocatalyst shows a minimal decrease from 96% (4th run) to 98% (1st run). Similarly, the degradation performance of OFL Antibiotics by the photocatalyst exhibits a minimal decrease from 96% (5th run) to 99% (1st run). The nanocomposite photocatalyst exhibited a high level of cycling performance, thereby confirming its good ability in cycling. Furthermore, Fig. 11 presents evidence of the structural stability of the catalyst that was synthesized. The FE-SEM analysis, as shown in Fig. 11A–C, reveals that the SEM images obtained before and after the photodegradation investigation show a similarity result. The X-ray diffraction (XRD) spectra (Fig. 11D) indicates that the sample morphology of both the fresh and used CDs/ZnO-H400 is comparable, suggesting their stability. This observation is further supported by the similarity in the XRD spectra before and after the photocatalytic process, as well as the FTIR spectra (Fig. 11E).

## Conclusion

Carbon dots from coconut water have been synthesized using hydrothermal methods, and mixed with Zinc Oxide (ZnO) using the same method, after being powdered, the powder is put into the calcination process at a temperature of 400 °C and takes 4 h to become the final powder. In this experiment, CD/ZnO-H400 showed a promising catalyst to reduce the RR141 and OFL antibiotics. The study also investigated the photocatalytic performance under sunlight and revealed a maximum degradation efficiency of 98% for RR 141 and 96% for the OFL Antibiotic. The decay of pollutants through photolysis follows the first order reaction. The optimum conditions of catalyst powder to reduced the pollutant show pH 7, in the 75 mg of catalyst with 5 ppm of RR141 and pH 7, 75 mg of catalyst in the 5 ppm of OFL. The CD/ZnO-H400 shows outstanding stability and high performance even after five cycles, which shows the potential for profitable cycle capabilities. Current studies show that the photocatalytic CDs/ZnO-H400 has potential for the environment.

## Data Availability

All data in this research are available from the corresponding author by reasonable request.

## References

[1] Chankhanittha T, Nanan S (2021). Visible-light-driven photocatalytic degradation of ofloxacin (OFL) antibiotic and Rhodamine B (RhB) dye by solvothermally grown ZnO/Bi_2_MoO_6_ heterojunction. J. Colloid Interface Sci..

[2] Jayarambabu N, Kumari BS, Rao KV, Prabhu YT (2014). Germination and growth characteristics of mungbean seeds (*Vigna radiata* L.). Int. J. Recent Technol. Eng..

[3] Vijayalakshmi U, Chellappa M, Anjaneyulu U, Manivasagam G, Sethu S (2016). Influence of coating parameter and sintering atmosphere on the corrosion resistance behavior of electrophoretically deposited composite coatings. Mater. Manuf. Process..

[4] Kakarndee S, Nanan S (2018). Journal of environmental chemical engineering SDS capped and PVA capped ZnO nanostructures with high photocatalytic performance toward photodegradation of reactive red (RR141) azo dye. J. Environ. Chem. Eng..

[5] Senasu T, Narenuch T, Wannakam K, Chankhanittha T, Nanan S (2020). Solvothermally grown BiOCl catalyst for photodegradation of cationic dye and fluoroquinolone-based antibiotics. J. Mater. Sci. Mater. Electron..

[6] Senasu T, Hemavibool K, Nanan S (2018). Hydrothermally grown CdS nanoparticles for photodegradation of anionic azo dyes under UV-visible light irradiation. RSC Adv..

[7] Amer WA, Al-Saida B, Ayad MM (2019). Rational design of a polypyrrole-based competent bifunctional magnetic nanocatalyst. RSC Adv..

[8] Velanganni S, Pravinraj S, Immanuel P, Thiruneelakandan R (2018). Nanostructure CdS/ZnO heterojunction configuration for photocatalytic degradation of Methylene blue. Phys. B Condens. Matter..

[9] Chankhanittha T, Somaudon V, Watcharakitti J, Piyavarakorn V, Nanan S (2020). Performance of solvothermally grown Bi_2_MoO_6_ photocatalyst toward degradation of organic azo dyes and fluoroquinolone antibiotics. Mater. Lett..

[10] Juabrum S, Chankhanittha T, Nanan S (2019). Hydrothermally grown SDS-capped ZnO photocatalyst for degradation of RR141 azo dye. Mater. Lett..

[11] Chankhanittha T, Watcharakitti J, Nanan S (2019). PVP-assisted synthesis of rod-like ZnO photocatalyst for photodegradation of reactive red (RR141) and Congo red (CR) azo dyes. J. Mater. Sci. Mater. Electron..

[12] Benes H, Cerna R, Durackova A (2016). Utilization of natural oils for decomposition of polyurethanes. J. Polym. Environ..

[13] Boukir A, Fellak S, Doumenq P (2019). Structural characterization of *Argania spinosa* Moroccan wooden artifacts during natural degradation progress using infrared spectroscopy (ATR-FTIR) and X-ray diffraction (XRD). Heliyon..

[14] Djunaidi M (2020). Gold imprinted adsorption based on eugenol. J. Phys. Conf. Ser..

[15] Gopinath V, Priyadarshini S, Al-Maleki AR, Alagiri M, Yahya R, Saravanan S, Vadivelu J (2016). In vitro toxicity, apoptosis and antimicrobial effects of phyto-mediated copper oxide nanoparticles. RSC Adv..

[16] Li Y, Kong D, Wu H (2018). Comprehensive chemical analysis of the flower buds of five *Lonicera* species by ATR-FTIR, HPLC-DAD, and chemometric methods. Rev. Bras. Farmacogn..

[17] Razali N, Conte M, McGregor J (2019). The role of impurities in the La_2_O_3_ catalysed carboxylation of crude glycerol. Catal. Lett..

[18] Rehman A, Eze V, Resul M, Harvey A (2019). A kinetic study of Zn halide/TBAB-catalysed fixation of CO_2_ with styrene oxide in propylene carbonate. Green Process Synth..

[19] Rozali M, Ahmad Z, Isa M (2015). Interaction between carboxy methylcellulose and salicylic acid solid biopolymer electrolytes. Adv. Mater. Res..

[20] Khanchandani S, Kundu S, Patra A, Ganguli AK (2012). Shell thickness dependent photocatalytic properties of ZnO/CdS core-shell nanorods. J. Phys. Chem. C..

[21] Lops C, Ancona A, Di Cesare K, Dumontel B, Garino N, Canavese G, Hernandez S, Cauda V (2019). Sonophotocatalytic degradation mechanisms of Rhodamine B dye via radicals generation by micro- and nano-particles of ZnO. Appl. Catal. B Environ..

[22] Shenoy S, Jang E, Park TJ, Gopinath CS, Sridharan K (2019). Cadmium sulfide nanostructures: Influence of morphology on the photocatalytic degradation of erioglaucine and hydrogen generation. Appl. Surf. Sci..

[23] Zhang J, Fang J, Ye X, Guo Z, Liu Y, Song Q, Zheng S, Chen X, Wang S, Yang S (2019). Visible photoactivity and antiphotocorrosion performance of CdS photocatalysts by the hybridization of N-substituted carboxyl group polyaniline. Appl. Surf. Sci..

[24] Mahmoodi NM, Abdi J, Taghizadeh M, Taghizadeh A, Hayati B, Shekarchi AA, Vossoughi M (2019). Activated carbon/metalorganic framework nanocomposite: Preparation and photocatalytic dye degradation mathematical modeling from wastewater by least squares support vector machine. J. Environ. Manag..

[25] Chankhanittha T, Somaudon V, Watcharakitti J, Nanan S (2021). Solar light-driven photocatalyst based on bismuth molybdate (Bi_4_MoO_9_) for detoxification of anionic azo dyes in wastewater. J. Mater. Sci. Mater. Electron..

[26] Chankhanittha T, Yenjai C, Nanan S (2021). Utilization of formononetin and pinocembrin from stem extract of *Dalbergia parviflora* as capping agents for preparation of ZnO photocatalysts for degradation of RR141 azo dye and ofloxacin antibiotic. Catal. Today..

[27] Chankhanittha T, Komchoo N, Senasu T, Piriyanon J, Youngme S, Hemavibool K, Nanan S (2021). Silver decorated ZnO photocatalyst for effective removal of reactive red azo dye and ofloxacin antibiotic under solar light irradiation. Colloids Surf. A Physicochem. Eng. Asp..

[28] Sansenya T, Masri N, Chankhanittha T, Senasu T, Piriyanon J, Mukdasai S, Nanan S (2022). Hydrothermal synthesis of ZnO photocatalyst for detoxification of anionic azo dyes and antibiotic. J. Phys. Chem. Solids..

[29] Narenuch T, Senasu T, Chankhanittha T, Nanan S (2021). Sunlight-active BiOI photocatalyst as an efficient adsorbent for the removal of organic dyes and antibiotics from aqueous solutions. Molecules..

[30] Hemavibool K, Sansenya T, Nanan S (2022). Enhanced photocatalytic degradation of tetracycline and oxytetracycline antibiotics by BiVO_4_ photocatalyst under visible light and solar light irradiation. Antibiotics..

[31] Yao Y, Zhang Y, Shen M, Li W, Xia W (2020). The facile synthesis and enhanced photocatalytic properties of ZnO@ZnS modified with Ag0 via in-situ ion exchange. Colloids Surf. A Physicochem. Eng. Asp..

[32] Beura R, Pachaiappan R, Paramasivam T (2021). Photocatalytic degradation studies of organic dyes over novel Ag-loaded ZnO graphene hybrid nanocomposites. J. Phys. Chem. Solids..

[33] Ha LPP, Vinh THT, Thuy NTB, Thi CM, Van Viet P (2021). Visible-light-driven photocatalysis of anisotropic silver nanoparticles decorated on ZnO nanorods: Synthesis and characterizations. J. Environ. Chem. Eng..

[34] Toe MZ, Le AT, Han SS, Yaacob KAB, Pung SY (2020). Silver nanoparticles coupled ZnO nanorods array prepared using photo-reduction method for localized surface plasmonic effect study. J. Cryst. Growth..

[35] Hassani A, Faraji M, Eghbali P (2020). Facile fabrication of mpg-C_3_N_4_/Ag/ZnO nanowires/Zn photocatalyst plates for photodegradation of dye pollutant. J. Photochem. Photobiol. A Chem..

[36] Wang W, Zhang D, Ji Z, Shao D, Sun P, Duan J (2021). High efficiency photocatalytic degradation of indoor formaldehyde with silver-doped ZnO/g-C_3_N_4_ composite catalyst under the synergistic effect of silver plasma effect and heterojunction. Opt. Mater..

[37] Iqbal S, Ahmad N, Javed M, Qamar MA, Bahadur A, Ali S, Ahmad Z, Irfan RM, Liu G, Akbar MB (2021). Designing highly potential photocatalytic comprising silver deposited ZnO NPs with sulfurized graphitic carbon nitride (Ag/ZnO/S-g-C_3_N_4_) ternary composite. J. Environ. Chem. Eng..

[38] Du C, Song J, Tan S, Yang L, Yu G, Chen H, Zhou L, Zhang Z, Zhang Y, Su Y (2021). Facile synthesis of Z-scheme ZnO/Ag/Ag_3_PO_4_ composite photocatalysts with enhanced performance for the degradation of ciprofloxacin. Mater. Chem. Phys..

[39] Iqbal S, Bahadur A, Ali S, Ahmad Z, Javed M, Irfan RM, Ahmad N, Qamar MA, Liu G, Akbar MB (2021). Critical role of the heterojunction interface of silver decorated ZnO nanocomposite with sulfurized graphitic carbon nitride heterostructure materials for photocatalytic applications. J. Alloys Compd..

[40] Ye W, Jiang Y, Liu Q, Xu D, Zhang E, Cheng X, Wan Z, Liu C (2022). The preparation of visible light-driven ZnO/Ag_2_MoO_4_/Ag nanocomposites with effective photocatalytic and antibacterial activity. J. Alloys Compd..

[41] Mohanty L, Pattanayak DS, Dash SK (2021). An efficient ternary photocatalyst Ag/ZnO/g-C_3_N_4_ for degradation of RhB and MG under solar radiation. J. Indian Chem. Soc..

[42] Badán JA, Jauregui G, Navarrete-Astorga E, Henríquez R, Jiménez FM, Ariosa D, Dalchiele EA (2021). Solid-state thermal dewetted silver nanoparticles onto electrochemically grown self-standing vertically aligned ZnO nanorods for three-dimensional plasmonic nanostructures. Ceram. Int..

[43] Farooq M, Shujah S, Tahir K, Nazir S, Khan AU, Almarhoon ZM, Jevtovic V, Al-Shehri HS, Hussain ST, Ullah A (2022). Ultra efficient 4-Nitrophenol reduction, dye degradation and Cr(VI) adsorption in the presence of phytochemical synthesized Ag/ZnO nanocomposite: A view towards sustainable chemistry. Inorg. Chem. Commun..

[44] Muñoz-Fernandez L, Gomez-Villalba LS, Miloševic O, Rabanal ME (2022). Influence of nanoscale defects on the improvement of photocatalytic activity of Ag/ZnO. Mater. Charact..

[45] Harinee S, Muthukumar K, James RA, Arulmozhi M, Dahms HU, Ashok M (2022). Bio-approach ZnO/Ag nano-flowers: Enhanced photocatalytic and photoexcited anti-microbial activities towards pathogenic bacteria. Mater. Today Sustain..

[46] Lee Y, Fujimoto T, Yamanaka S (2022). Characterization of submicro-sized Ag/ZnO particles generated using the spray pyrolysis method. Adv. Powder Technol..

[47] Senasu T, Nanan S (2017). Photocatalytic performance of CdS nanomaterials for photodegradation of organic azo dyes under artificial visible light and natural solar light irradiation. J. Mater. Sci. Mater. Electron..

[48] Christy EJS, Amalraj A, Rajeswari A, Pius A (2021). Enhanced photocatalytic performance of Zr(IV) doped ZnO nanocomposite for the degradation efficiency of different azo dyes. Environ. Chem. Ecotoxicol..

[49] Senasu T, Youngme S, Hemavibool K, Nanan S (2021). Sunlight-driven photodegradation of oxytetracycline antibiotic by BiVO_4_ photocatalyst. J. Solid State Chem..

[50] Jo WK, Kumar S, Isaacs MA, Lee AF, Karthikeyan S (2017). Cobalt promoted TiO_2_/GO for the photocatalytic degradation of oxytetracycline and Congo Red. Appl. Catal. B Environ..

[51] Shinde SG, Patil MP, Kim GD, Shrivastava VS (2020). Multi-doped ZnO photocatalyst for solar induced degradation of indigo carmine dye and as an antimicrobial agent. J. Inorganic Organometal. Polym. Mater..

[52] Samadi M, Zirak M, Naseri A, Kheirabadi M, Ebrahimi M, Moshfegh AZ (2019). Design and tailoring of one-dimensional ZnO nanomaterials for photocatalytic degradation of organic dyes: A review. Res. Chem. Intermed..

[53] Nugroho D, Keawprom C, Chanthai S, Oh W-C, Benchawattananon R (2022). Highly sensitive fingerprint detection under UV light on non-porous surface using starch-powder based luminol-doped carbon dots (N-CDs) from tender coconut water as a green carbon source. Nanomaterials.

[54] Nugroho D, Oh W-C, Chanthai S, Benchawattananon R (2022). Improving minutiae image of latent fingerprint detection on non-porous surface materials under UV light using sulfur doped carbon quantum dots from *Magnolia grandiflora* flower. Nanomaterials..

[55] Jiao Y, Gong X, Han H, Gao Y, Lu W, Liu Y, Xian M, Shuang S, Dong C (2018). Facile synthesis of orange fluorescence 1 carbon dots with excitation independent emission for pH sensing and cellular imaging. Anal. Chim. Acta..

[56] Gao D, Gao F, Kuang Q, Zha X, Zhang Z, Pan Y, Chai R, Jiao H (2022). Zinc germanate nanophosphors with persistent luminescence for multi-mode imaging of latent fingerprints. ACS Appl. Nano Mater..

[57] Koutsogiannis P, Thomou E, Stamatis H, Gournis D, Rudolf P (2020). Advances in fluorescent carbon dots for biomedical applications. Adv. Phys.-X..

[58] Zhang Q, Zhao Q, Fu M, Fan X, Lu H, Wang H, Zhang Y, Wang H (2018). Carbon quantum dots encapsulated in super small platinum nanocrystals core-shell architecture/nitrogen doped graphene hybrid nanocomposite for electrochemical biosensing of DNA damagebiomarker-8′-hydroxy-2′-deoxyguanosine. Anal. Chim. Acta.

[59] Macairan J-R, Jaunky DB, Naccache R, Piekny A (2019). Intracellular ratiometric temperature sensing using fluorescent carbon dots. Nanoscale Adv..

[60] Feng T, Ai X, Ong H, Zhao Y (2016). Dual-responsive carbon dots for tumor extracellular microenvironment triggered targeting and enhanced anticancer drug delivery. ACS Appl. Mater. Interfaces..

[61] Yan Y, Gong J, Chen J, Zeng Z, Huang W, Pu K, Liu J, Chen P (2019). Recent advances on graphene quantum dots: From chemistry and physics to applications. Adv. Mater..

[62] Han W, Li D, Kong Y, Liu W, Qin W, Wang S, Duan X (2023). High-performance photocatalytic peroxymonosulfate activation by carbon quantum dots via precise surface chemistry regulation: Insight into the structure–function relations. J. Colloid Interface Sci..

[63] Bartolomei, B., Corti, V. & Prato, M. Chiral carbon nanodots can act as molecular catalysts in chemical and photochemical reactions. *Angewandte Chemie* **135** (2023) 10.1002/anie.202305460 37334995

[64] Bartolomei B, Corti V, Prato M (2023). Chiral carbon nanodots can act as molecular catalysts in chemical and photochemical reactions. Angew. Chem. Int. Ed..

[65] Han M, Zhu S, Lu S, Song Y, Feng T, Tao S, Liu J, Yang B (2018). Recent progress on the photocatalysis of carbon dots: Classification, mechanism and applications. Nano Today..

[66] Dhenadhayalan N, Lin KC, Saleh TA (2020). Recent advances in functionalized carbon dots toward the design of efficient materials for sensing and catalysis applications. Small..

[67] Koli PB, Birari MD, Ahire SA, Shinde SG, Ingale RS, Patil IJ (2022). Ferroso-ferric oxide (Fe_3_O_4_) embedded g-C_3_N_4_ nanocomposite sensor fabricated by photolithographic technique for environmental pollutant gas sensing and relative humidity characteristics. Inorganic Chem. Commun..

[68] Shinde SG, Shrivastava VS (2020). Ni and Zn modified acid activated montmorillonite clay for effective removal of carbol fuchsin dye from aqueous solution. SN Appl. Sci..

